# Copper alumina @ poly (aniline-*co*-indole) nanocomposites: synthesis, characterization, electrical properties and gas sensing applications

**DOI:** 10.1039/d2ra02213c

**Published:** 2022-06-14

**Authors:** S. Sankar, Ajith George, M. T. Ramesan

**Affiliations:** Centre for Polymer Science and Technology, Department of Chemistry, University of Calicut Calicut University P.O. 673 635 Kerala India mtramesan@uoc.ac.in +91 4942400269 +91 4942401413

## Abstract

Poly(aniline-co-indole)/copper alumina (PANI-*co*-PIN/Cu–Al_2_O_3_) with excellent AC conductivity, dielectric properties, and ammonia gas detecting capabilities were synthesised *via in situ* chemical oxidative polymerization. The presence of Cu–O bonding vibrations and shift of some characteristic peaks in the Fourier transform infrared spectroscopy (FT-IR) revealed the successful encapsulation of Cu–Al_2_O_3_ nanoparticles in the copolymer. The XRD studies showed the crystalline peaks of Cu–Al_2_O_3_ in the PANI-*co*-PIN nanocomposites. The high-resolution transmission electron microscopy (HR-TEM) images confirmed the reinforcement of the inorganic moiety in the copolymer. The results from thermogravimetric analysis (TGA) showed that the inclusion of Cu–Al_2_O_3_ in the copolymer matrix greatly increases the thermal stability of PANI-*co*-PIN. The alternate current (AC) conductivity and dielectric properties of nanocomposites were higher than pure PANI-*co*-PIN. The improved electrical properties of nanocomposites were due to strong contact between the copolymer and metal oxide surfaces. The gas sensing properties of synthesized copolymer nanocomposites showed excellent sensitivity and response towards ammonia gas at room temperature. The PANI-*co*-PIN/5 wt% Cu–Al_2_O_3_ nanocomposite has the best gas sensing characteristics. The higher AC conductivity, dielectric properties and gas sensing characteristics of PANI-*co*-PIN/Cu–Al_2_O_3_ might be used to develop electrochemical sensing devices.

## Abstract 2

PANI-*co*-PIN/Cu–Al_2_O_3_ nanocomposites synthesised *via in situ* polymerization showed excellent electrical and NH_3_ gas sensing properties.

## Graphical abstract

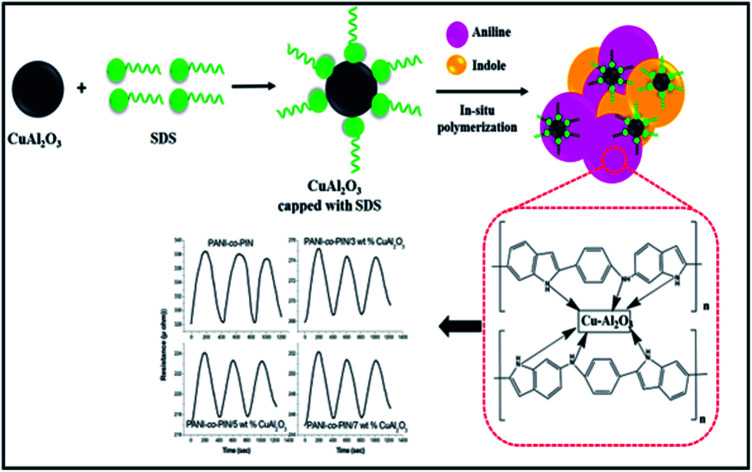

## Introduction

Metal oxide nanoparticles play a crucial role in several technological applications on account of their distinctive physical as well as chemical properties.^1–4^ The size of the nanoparticle is a key factor affecting its electrical, magnetic and thermal properties. Major examples of metal oxide nanoparticles are ZnO, CuO, TiO_2_ and Al_2_O_3_, *etc.* Integrating metal oxide nanoparticles and polymer matrixes improves the properties of polymers.^5,6^ The enhancement of properties arises from the interaction of metallic components with the organic moiety.^7^ Conducting polymer/metal oxide nanocomposites have superior electrical, thermal and optical properties, making them potential candidates for high-tech device manufacturing.^8–10^ The Al_2_O_3_ nanomaterial is inexpensive, thermally stable and has outstanding mechanical and dielectric properties compared to other metal oxide nanoparticles.^11^ The electrical conductivity of Al_2_O_3_ can be improved by dispersion strengthening, which happens when divalent ions such as copper occupy locations in the matrix. Thus, Cu–Al_2_O_3_ has better electrical conductivity, thermal characteristics and mechanical properties than other metal oxides.^12^

Polyaniline (PANI) is a fantastic conducting polymer because of its low-cost monomer, simple synthesis method, environmental stability and high electrical conductivity.^13,14^ However, the thermal properties of PANI are at rock bottom. The thermal properties of PANI can be amplified by blending or by copolymerization technique.^15^ The synthesis of copolymer nanocomposites enables us to eliminate the drawbacks of homopolymers by combining their excellent properties.

Conducting polymers and their blends are widely used as sensors due to their easy synthesis and the conjugated electronic structure.^16^ Ammonia gas is being used in many sectors, such as refrigeration, dyes, chemical plants, *etc.* Ammonia gas is harmful beyond a certain limit of exposure, so the need for an ammonia sensor is an essential requirement in the aforementioned industries. The conducting polymer nanocomposites have excellent ammonia gas sensing properties compared to their homopolymers. For instance, Yin *et al.* synthesized polypyrrole-rGO nanocomposites decorated by Cu-BTC having great potential for ammonia gas sensing.^17^ Majumdar *et al.* fabricated a chemically stable and low-cost ammonia gas sensor out of polypyrrole coated filter paper.^18^ The electron transport from ammonia gas to sensing materials regulates the sensing behaviour of conducting polymer-based nanocomposites. Generally, metal oxide nanoparticles can impart gas sensing to polymer composites.^19^ Dhingra *et al.* developed a PANI/ZnO nanocomposite that demonstrated strong ammonia sensitivity, however, the sensing material had a longer recovery time.^20^ Bandgar *et al.*^21^ developed a flexible PANI/α-Fe_2_O_3_ nanocomposite with high NH_3_ gas selectivity. In previous research,^22,23^ we found that PANI/Cu–Al_2_O_3_ and PIN/Cu–Al_2_O_3_ had excellent electrical, thermal and ammonia gas sensing properties. The synergistic interaction of Cu–Al_2_O_3_ with the polar entity of a conducting polymer is the basis of their superior properties. This motivates us to investigate the characteristics of PANI-*co*-PIN nanocomposite, including Cu–Al_2_O_3_ as a filler.

Various strategies for preparing conducting polymer nanocomposites are electrochemical method,^24^ mechanical mixing,^25^ solvent mixing^26^ and *in situ* chemical oxidative polymerization.^27^ The electrochemical approach is complicated since the particle size cannot be tailored. The use of hazardous solvents in solvent mixing is antithetical to green chemistry. *In situ* polymerization is simple, environmentally benign and provides the best interaction between polymer and metal oxide nanoparticles. The homogeneous dispersion of nanoparticles at the polymer interface causes the greatest contact. As a result, materials are endowed with extraordinary properties.^6,28^

No works have been reported on the synthesis and characterization of Cu–Al_2_O_3_ nanoparticles reinforced PANI-*co*-PIN copolymer nanocomposites. The intention of the conferred work is to synthesize PANI-*co*-PIN/Cu–Al_2_O_3_*via in situ* oxidation polymerization. Structural features of PANI-*co*-PIN/Cu–Al_2_O_3_ are evaluated by FT-IR, XRD and HR-TEM. Insights about the thermal stability of the material are investigated using TGA. The research investigates the impact of Cu–Al_2_O_3_ nanoparticles on the ammonia gas sensing characteristics, electrical conductivity and dielectric parameters of PANI-*co*-PIN/Cu–Al_2_O_3_.

## Experimental

### Materials and methods

The materials used for the synthesis of PANI-*co*-PIN/Cu–Al_2_O_3_ nanocomposites were monomers (aniline and indole), ferric chloride (FeCl_3_), sodium dodecyl sulphate (SDS), copper nitrate (Cu (NO_3_)_2_), aluminium nitrate (Al (NO_3_)_3_), urea, HCl and methanol were procured from Merck India. The solvent used for all the synthesis procedures in this work was distilled water. The copper alumina nanoparticles were synthesized by the thermochemical mode of synthesis as reported earlier.^29^

### Synthesis of poly (aniline-*co*-indole) copolymer

The copolymer was synthesised by the oxidative polymerization of an equimolar ratio of aniline and indole with ferric chloride as an oxidant. First, 1.0 M FeCl_3_ was dissolved in water and stirred for 15 min at 0 °C in a three-neck flask. Certain amounts of aniline and indole monomers were dissolved in an acid aqueous medium and these homogeneous solutions were slowly added from a dropping funnel to the FeCl_3_ solution at 0 °C. The copolymer formation was visible after a few minutes in the course of the reaction, reddish FeCl_3_ solution was turned to a dark green colour, indicating the consumption of monomers. This solution was permitted to polymerize by maintaining the ice-cold temperature and the stirring continued for 8 h. The unreacted monomers and oxidants were removed from the precipitated poly (aniline-*co*-indole) by washing several times with water and methanol and then dried in a vacuum oven at 60 °C for 24 h.

### Synthesis of poly(aniline-*co*-indole)/Cu–Al_2_O_3_ nanocomposites

Different contents of copper alumina reinforced PANI-*co*-PIN nanocomposites were synthesized by *in situ* polymerization of aniline and indole in an aqueous acidic condition using ferric chloride as an oxidizing agent. Cu–Al_2_O_3_ nanoparticles (3, 5 and 7 wt%) were mixed with SDS in distilled water. This nanofluid was then added to the mixture of aniline and indole and ultrasonicated for half an hour. The FeCl_3_ solution (1 M) was then added dropwise to the homogeneous solution of monomers with constant stirring for half an hour. The copolymerization was carried out for a period of 8 h at a temperature of 0 °C. The precipitated fine green coloured particles were filtered from the reaction mixture and thoroughly washed with distilled water and methanol several times to remove the unreacted monomers and oxidant. The nanocomposite was finally dried in a vacuum oven at 60 °C for 24 h.

### Instrumentation

FT-IR analysis of PANI-*co*-PIN/Cu–Al_2_O_3_ and Cu–Al_2_O_3_ composites were measured in the range of 4000 to 450 cm^−1^ in a JASCO 4100 FT-IR spectrometer using the KBr pellet method. The XRD profile of Cu–Al_2_O_3_, PANI-*co*-PIN and their nanocomposites was carried out on the Bruker D8, advanced X-ray diffractometer. The samples were scanned in the range of 20 to 80° at a scanning rate of 5° min^−1^. TEM images were photographed in JEOL, JEM–2100 HR (Japan) respectively. TGA was recorded in Hitachi STA7200 respectively at a heating rate of 10 °C min^−1^. The variation of AC conductivity and dielectric traits with frequency (10^2^ to 10^6^ Hz) was measured in the Hioki 3570 Model. The gas sensing traits were analysed by passing ammonia gas into the glass chamber containing PANI-*co*-PIN/Cu–Al_2_O_3_ nanocomposites. The concentration of ammonia gas used was 100 ppm and measurements were conducted at room temperature. The sensitivity was expressed in terms of resistance and was calculated using a digital multimeter. The sensor response of synthesized nanocomposites was calculated by
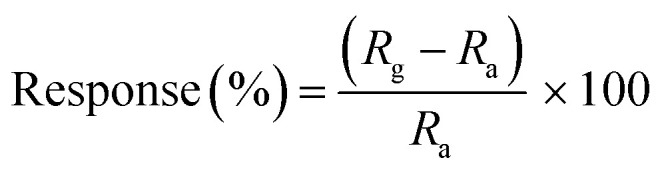
where *R*_g_ and *R*_a_ are the resistance of PANI-*co*-PIN/Cu–Al_2_O_3_ nanocomposites measured in the presence of NH_3_ gas and air, respectively. Entire samples were pelletized into a circular disc prior to conductivity and gas sensing measurements.

## Results and discussion

### Fourier Transform infrared spectroscopy (FT-IR)

**Fig. 1:**
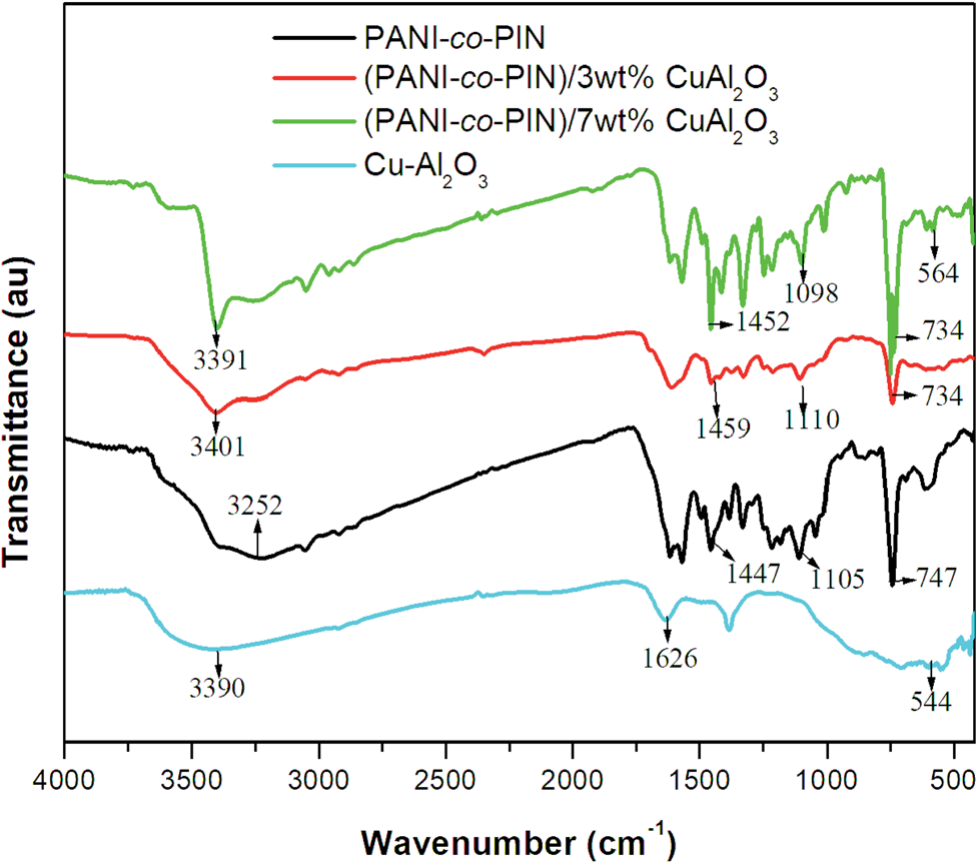
FT-IR spectrum of pure Cu–Al_2_O_3_, copolymer and its nanocomposites.

The structural elucidation of PANI-*co*-PIN/Cu–Al_2_O_3_ and interactions between organic and inorganic moieties are studied using FT-IR spectroscopy. The FTIR spectrum of pure Cu–Al_2_O_3_, PANI-*co*-PIN and their nanocomposites are displayed in Fig. 1. Our previous study on PIN-based materials reported that characteristic bands at 3411, 1561, 1470, 1109 and 747 cm^−1^ are the resultant of N–H stretch, C=C stretch, C=C bend, C–N deformation and C–H deformation respectively.^23^ Similarly, the FT-IR studies on PANI were reported to show peaks at 3438 cm^−1^(N–H stretch), 1482 cm^−1^ (C=C stretch) and 1306 cm^−1^ (C–N stretch of 2° amine).^22^ The copolymerization of aniline and indole has been validated by the FT-IR spectrum, with distinctive PANI and PIN peaks present in the FT-IR spectra of copolymer and nearly all bands are shifted to a lower wavenumber. This shift in IR peaks is due to the polar–polar interaction between the metal oxide nanoparticles and the copolymer segments. For example, the N–H stretch of the copolymer is obtained at 3252 cm^−1^. The Cu–O stretching, O–H stretching and bending vibrations are observed in the FT-IR spectrum of Cu–Al_2_O_3_ nanoparticles at 544 cm^−1^, 3399 cm^−1^ and 1626 cm^−1^ respectively. The characteristic Cu–O band is clearly observed in the FT-IR spectrum of PANI-*co*-PIN/CuAl_2_O_3_ nanocomposite at 564 cm^−1^ with the characteristic bands of copolymer indicating the successful polymerization of nanoparticles inside the copolymer segments. It can be seen from the figure that the N–H vibration of the copolymer (3252 cm^−1^) is found to be shifted to a higher wavenumber at 3401 cm^−1^ by the insertion of nanoparticles into the copolymer. These shifts in peaks suggest the strong interaction between PANI-*co*-PIN and Cu–Al_2_O_3_ nanoparticles.

### X-Ray diffraction (XRD) analysis

**Fig. 2:**
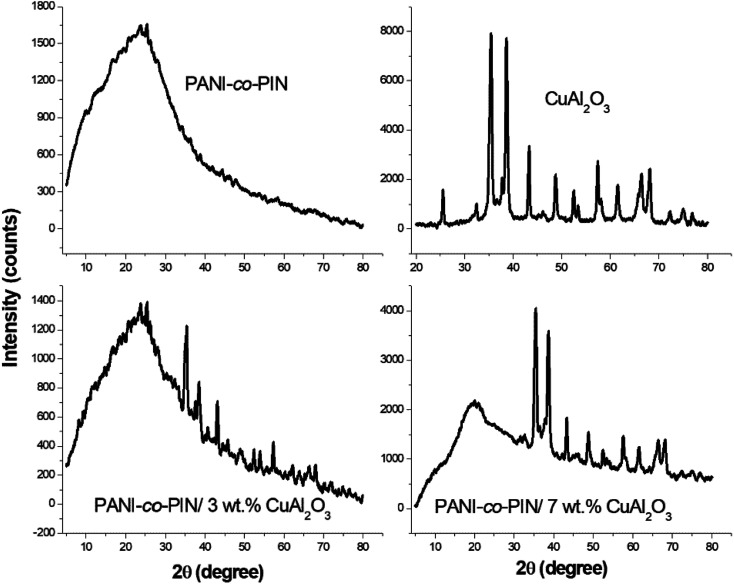
XRD of copolymer, Cu–Al_2_O_3_ and copolymer/Cu–Al_2_O_3_ nanocomposites.

The variations in crystallinity and order of PANI-*co*-PIN reinforcement of Cu–Al_2_O_3_ nanocomposites are studied using the XRD profile. The XRD profiles of Cu–Al_2_O_3_, PANI-*co*-PIN and PANI-*co*-PIN/Cu–Al_2_O_3_ nanocomposites at 3 and 7 wt% are given in Fig. 2. The crystalline peaks are spotted at 2*θ* values of 25.35°, 32.52°, 35.35°, 38.67°, 43.35°, 48.68°, 52.50°, 57.64°, 60.73°, 66.09° and 68.39° which point out the perfect ordered nature of Cu–Al_2_O_3_ nanoparticles. On the other hand, PANI-*co*-PIN has little or no long-range order. The crystalline peaks in the XRD of PANI-*co*-PIN/Cu–Al_2_O_3_ nanocomposite manifest the improvement in long-range order of the copolymer with reinforcement of Cu–Al_2_O_3_ nanoparticles.

This finding confirms the inclusion of Cu–Al_2_O_3_ nanoparticles within the copolymer. Moreover, the intensity of crystalline peaks of nanocomposites is enhanced with an increase in the amount of filler particles. The existence of strong interfacial contact between nitrogen in the copolymer and Cu–O phase in Cu–Al_2_O_3_ nanoparticles is confirmed by these increase in long-range order.^24^

### High resolution transmission electron microscopy (HR-TEM)

**Fig. 3:**
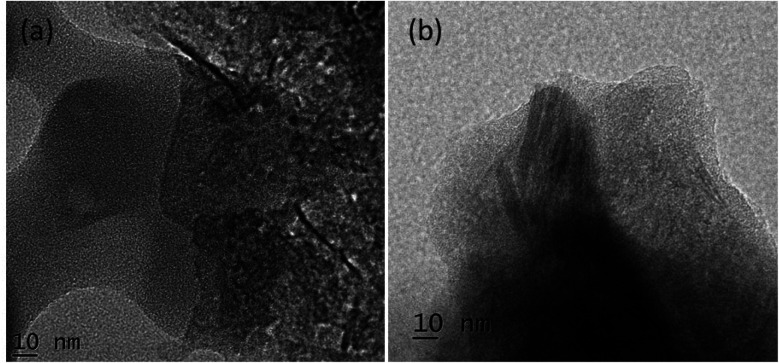
HR-TEM images of copolymer with (a) 5 and (b) 7 wt% of Cu–Al_2_O_3_.

The changes in the morphology and particle size of PANI-*co*-PIN with the addition of Cu–Al_2_O_3_ nanoparticles are examined using HR-TEM. HR-TEM images of PANI-*co*-PIN/Cu–Al_2_O_3_ nanocomposites with 5 and 7 wt% loadings are presented in Fig. 3. The Cu–Al_2_O_3_ nanoparticles in the copolymer appear as dark patches in HR-TEM pictures. The HR-TEM of PANI-*co*-PIN/Cu–Al_2_O_3_ nanocomposites confirms the encapsulation of Cu–Al_2_O_3_ within the PANI-*co*-PIN matrix. Among the nanocomposites, better distribution of Cu–Al_2_O_3_ nanomaterial is observed in PANI-co-PIN/5 wt% Cu–Al_2_O_3_.

The aggregation of nanofillers observed at higher loading is due to the poor polymer–filler interaction rather than the filler–filler interaction. The particle-to-particle spacing in the composite reduces as the loading increases, resulting in nanoparticle aggregation. The average size of the metal oxide particles in the copolymer is found to be 40 nm.

### Thermogravimetric analysis (TGA)

**Fig. 4:**
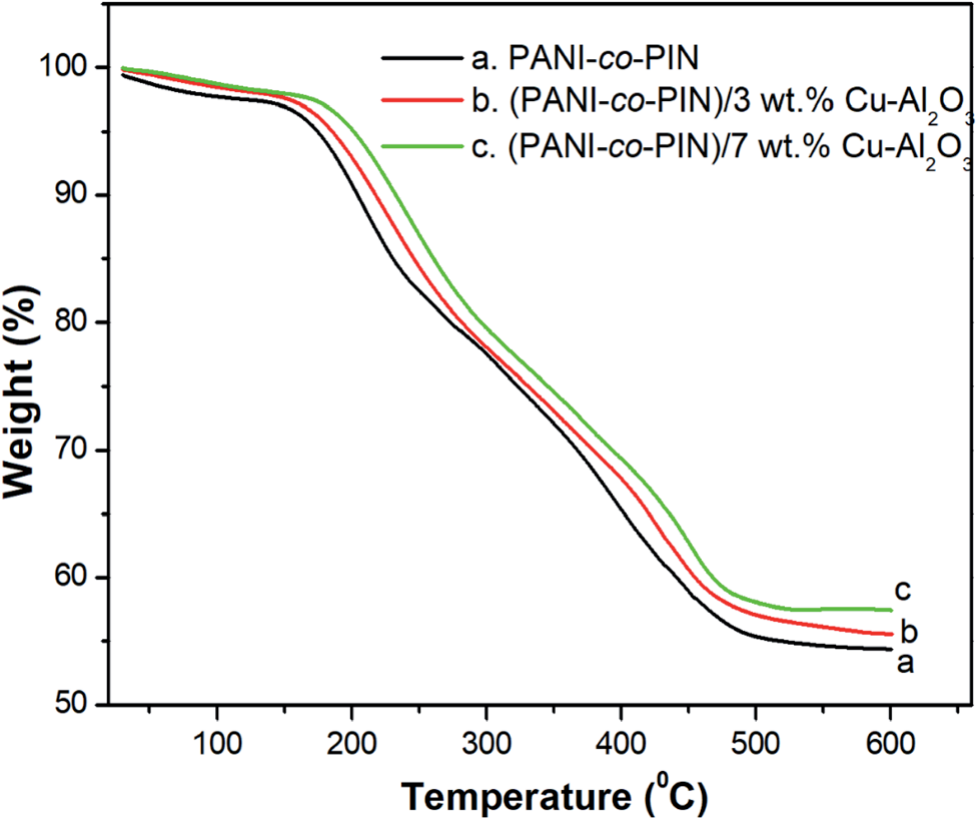
TGA of copolymer with different contents of Cu–Al_2_O_3_.

The variations in thermal stability of PANI-*co*-PIN with reinforcement of Cu–Al_2_O_3_ are studied using TGA. Fig. 4 demonstrates the TGA graph of PANI-*co*-PIN and their nanocomposites with Cu–Al_2_O_3_ as nanofillers at different loadings. It can be seen from the figure that all the samples exhibit three stages of thermal decomposition. The minor degradation below 130 °C is assigned to the evaporation of volatile contaminants from the copolymer. The major degradation from 140 to 285 °C depicts the decomposition of unreacted monomer and PANI segment. After that, the residual copolymer degrades from 320 to 510 °C is the carbon-containing residue. It is evident from the figure that the thermal degradation temperature of the nanocomposite is higher than the pure copolymer and the thermal stability increases with an increase in the concentration of copper alumina nanoparticles. The enhanced thermal stability of the copolymer nanocomposite is due to the strong interaction between nanoparticles and the copolymer segments. At 600 °C, the final char residue for copolymer is 54.25%, whereas the final char residue for nanocomposite with 3 and 7 wt% Cu–Al_2_O_3_ nanoparticles is 55.68% and 57.57%, respectively. This is another evidence of the increased thermal stability of the prepared copolymer nanocomposites. This result is in good agreement with the earlier work.^12^

### Alternating current conductivity

**Fig. 5:**
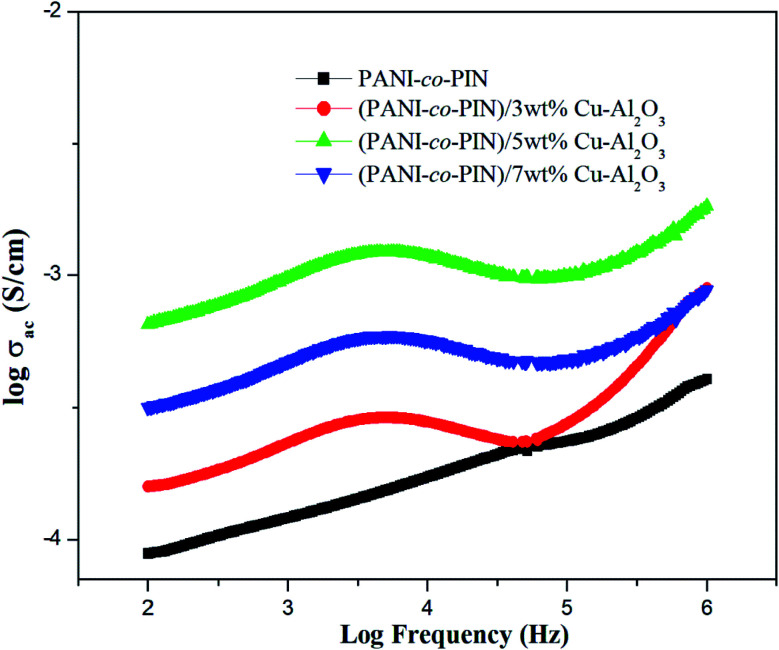
Variation of AC conductivity of copolymer/Cu–Al_2_O_3_ nanocomposites.

Fig. 5 shows the room temperature AC conductivity profile of PANI-*co*-PIN and PANI-*co*-PIN/Cu–Al_2_O_3_ nanocomposites at different frequencies. Three distinct areas are found in the AC conductivity profile of all materials. The initial area of the graph (low frequency) has a linear character, which is due to the disordered structure of polymeric materials. There is a decrease in AC conductivity that is observed beyond 10^4^ Hz. This observation might be due to the delayed movement of charges caused by electrode polarization. The last zone is characterized by substantial inflation and is located between 10^5^ and 10^6^ Hz. This hike in electrical conductivity is the result of polarizations, grain boundaries, dopants and imperfections.^30^ The irregularity of the bare copolymer is minimized by the inclusion of nanofillers hence AC conductivity of polymer nanocomposites is higher than the copolymer. The regular and ordered nature aids the swift transport of charges through the network. This result is in good agreement with the functionalized graphene oxide reinforced polymer composites.^31^

The 5 wt% composite has the most regularity and ordered arrangements (in good agreement with the TEM analysis) and hence has the highest conductivity among other copolymer nanocomposites. The agglomeration of nanoparticles at higher loadings reduces the regularity of the polymeric materials. As a result, the conductivity of 7 wt% composite is lower than that of the PANI-*co*-PIN/5 wt% Cu–Al_2_O_3_ nanocomposite.

### Dielectric constant

**Fig. 6:**
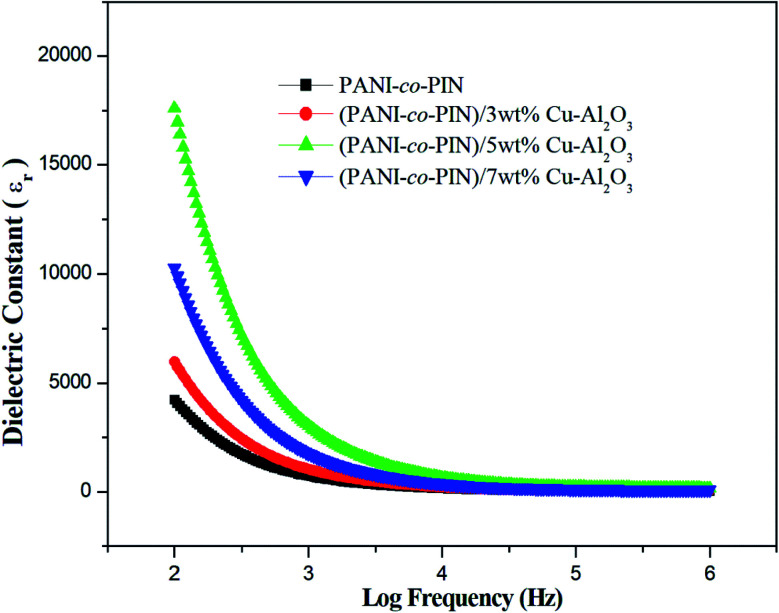
Dielectric constant of copolymer/Cu–Al_2_O_3_ nanocomposites.

The dielectric constant measures the capacity of a material to get polarised in the presence of an electric field. Fig. 6 shows the room temperature frequency dependence of PANI-*co*-PIN/Cu–Al_2_O_3_ nanocomposites. Higher dielectric constant values at low-frequency ranges account for the aggregation of loosely bound charge carriers at grain boundaries. While at higher frequencies, lower values are observed, which points to the dielectric relaxation mechanism. The PANI-*co*-PIN/Cu–Al_2_O_3_ nanocomposites show better dielectric properties than the bare copolymer because charge carriers (polarons and bipolarons) are formed within the material.^32^

Among all samples, the PANI-*co*-PIN/5wt% Cu–Al_2_O_3_ nanocomposite possesses the maximum dielectric constant values as polarization effects are excellent here due to the efficient alliance between macromolecules and nanofillers. With further incorporation of nanofillers, the interfacial polarisation decreases due to curtailed interaction between the polymer and metal oxide nanofillers (evident from TEM images).

### Dielectric loss tangent (tan *δ*)

**Fig. 7:**
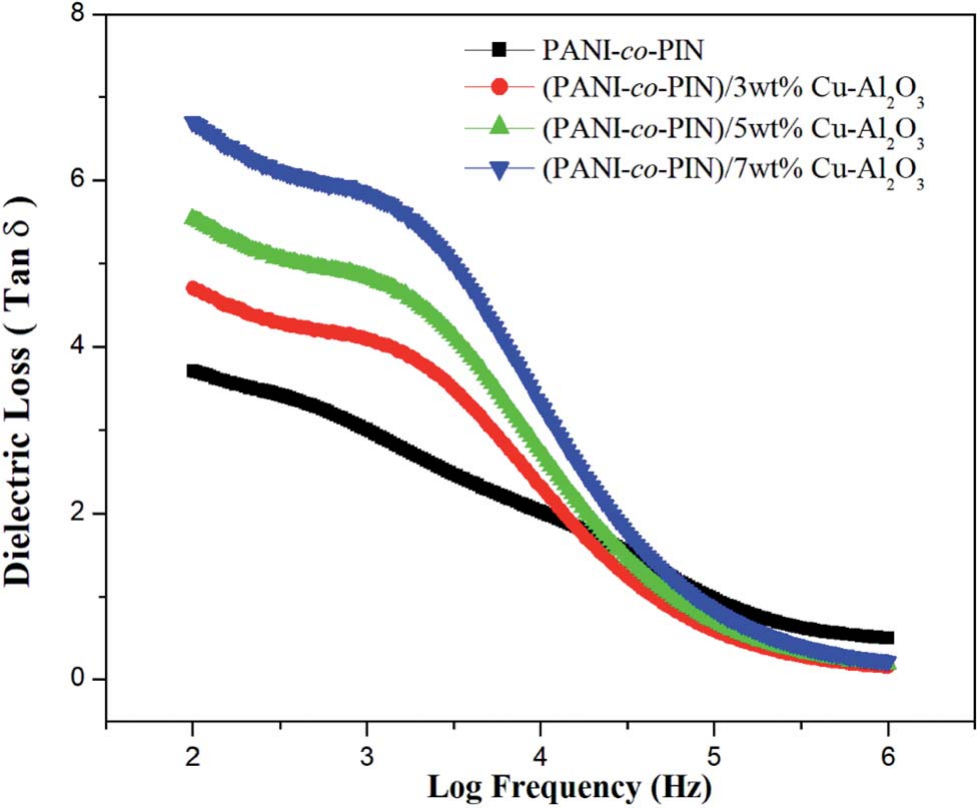
Dielectric loss tangent of copolymer/Cu–Al_2_O_3_ nanocomposites.

The dielectric loss tangent *versus* frequency graph is exhibited in Fig. 7. The tan *δ* value at low frequency denotes frozen dipoles in the material. While the constant area in the plot at higher frequency denotes the electronic and atomic polarization. Moreover, relaxation peaks are observed for entire samples at intermediate frequencies. The relaxation behaviour can well be explained using the Maxwell–Wagner–Sillars polarization effect. The orientation of polar moieties inside the copolymer and their nanocomposites induces energy dissipation in the form of heat under the effect of frequency. At a particular frequency, elastic restoring force makes the polar moieties confine at the equilibrium position. Lowering of relaxation time is observed with the reinforcement of Cu–Al_2_O_3_ nanofillers. The higher conductivity of nanocomposites can be ascribed to this behaviour. The interfacial polarizations generally decide the dielectric loss of material. The excellent tan *δ* value and minimum relaxation time are observed for PANI-*co*-PIN/5wt% Cu–Al_2_O_3_ nanocomposites. Excellent interfacial interaction of 5 wt% nanocomposite is responsible for their maximum tan *δ* value. Furthermore, when filler particle addition is greater than 5 wt%, dielectric values are reduced due to a lack of interaction between the copolymer and filler interfaces.

### Gas sensing properties

**Fig. 8:**
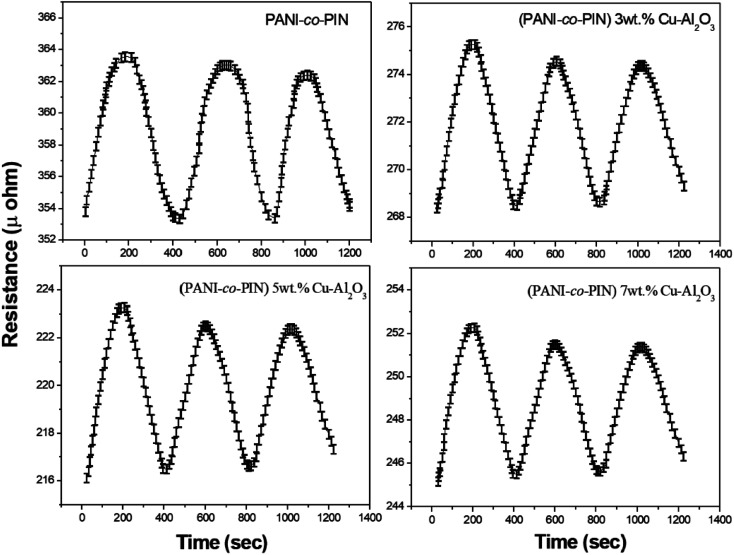
Gas sensing response for NH_3_ gas of copolymer with various contents of Cu–Al_2_O_3_.

**Fig. 9:**
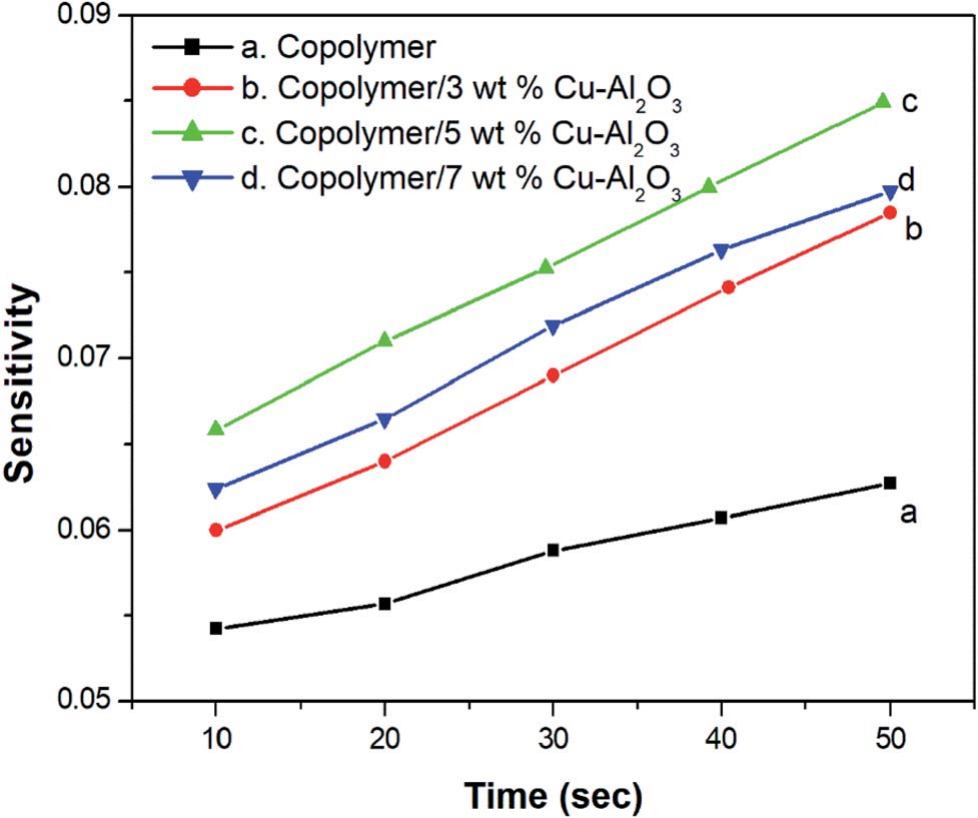
Change in gas sensitivity of copolymer with different contents of Cu–Al_2_O_3_.

Fig. 8 indicates the NH_3_ gas sensing characteristics of PANI-*co*-PIN and PANI-*co*-PIN/Cu–Al_2_O_3_ nanocomposites. The surface reaction between copolymeric fragments and NH_3_ gas can be ascribed to the variation in resistance of sensor materials by the passage of NH_3_ gas. The reversibility and recovery of sensing materials are confirmed by the wavy nature of the resistance *versus* time graph.^33^ This reversibility of PANI-*co*-PIN and PANI-*co*-PIN/Cu–Al_2_O_3_ nanocomposites can be well explained with the aid of Langmuir–Hinshelwood mechanism.^34^ When compared to PANI-*co*-PIN, the resistance value of PANI-*co*-PIN/Cu–Al_2_O_3_ nanocomposites drops significantly. A p–n junction is formed when Cu–Al_2_O_3_ is added to PANI-*co*-PIN. The charge carriers present within the p–n junction account for the reduced resistance of PANI-*co*-PIN/Cu–Al_2_O_3_ nanocomposites. The lower resistance is observed for PANI-*co*-PIN/5 wt% Cu–Al_2_O_3_ nanocomposites owing to the uniform dispersion of nanofillers in the copolymer matrix. The better dispersion of nanomaterial into the copolymer facilitates charge transfer through the depletion layer.^35^ Due to the clustering of nanofillers in the PANI-*co*-PIN, greater resistance is visible at higher nanofiller loading. The change in gas sensitivity of the copolymer at various loadings of nanoparticles at room temperature is given in Fig. 9. For all samples, the sensitivity measured on exposure to NH_3_ gas increases linearly with time. The PANI-*co*-PIN has a much lower sensitivity than PANI-*co*-PIN/Cu–Al_2_O_3_ nanocomposites. The better NH_3_ gas sensitivity of polymer nanocomposites is associated with the interfacial association between the polymer and nanofillers. In analogy with the resistance *versus* time graph, PANI-*co*-PIN/5 wt% Cu–Al_2_O_3_ nanocomposites show better sensitivity due to the consistent dispersion of nanofillers over the polymer. At higher loadings, sensitivity toward NH_3_ gas is found to be reduced. The assemblage of nanoparticles significantly minimizes space within the polymer, thereby preventing surface reaction with NH_3_ gas. When compared to PIN/Cu–Al_2_O_3_ nanocomposites, copolymer nanocomposites show superior gas sensing properties.^23^ PANI-*co*-PIN/Cu–Al_2_O_3_ has a stronger initial gas sensitivity response than PANI/Cu–Al_2_O_3_ nanocomposites.^22^

### Gas sensing mechanism

**Scheme 1:**
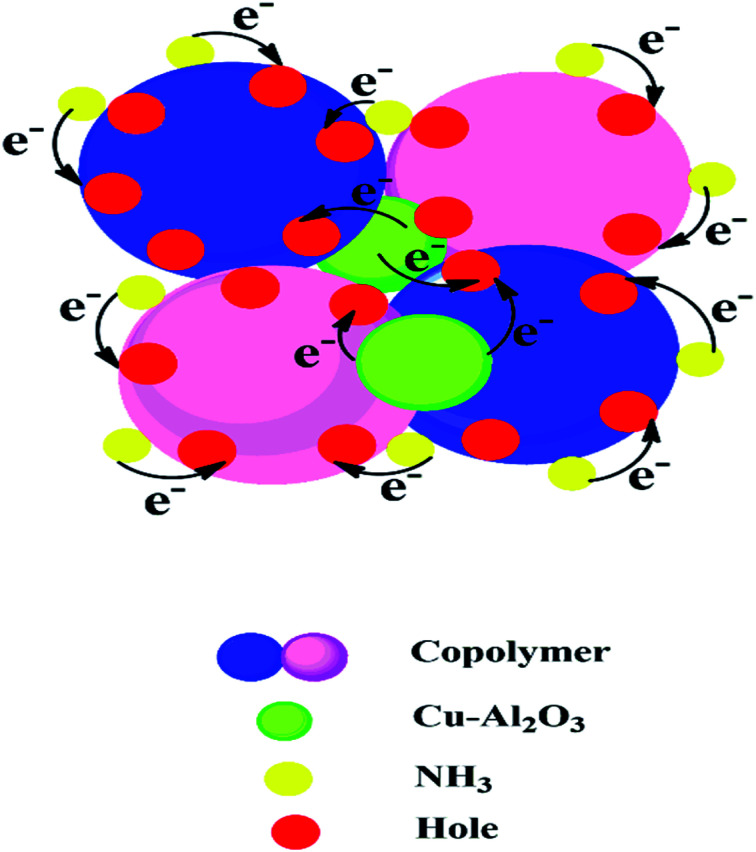
Gas sensing mechanism of PANI-*co*-PIN/Cu–Al_2_O_3_ nanocomposites.

The gas sensing mechanism of copolymer nanocomposites is given in Scheme 1. In the sensing chamber, ammonia gas molecules are physisorbed on the N centres of PANI-*co*-PIN. The hole density in the copolymer is compensated by the electron donating nature of the target gas. In nanocomposites, the transfer of electrons from Cu–Al_2_O_3_ nanofillers to the copolymer generates a positive depletion layer that reduces the enthalpy of physisorption of the target gas. The higher sensitivity of 5 wt% nanocomposite can be attributed to the swift transfer of electrons from nanofillers to copolymer aided by the homogenous dispersion of nanofillers. A similar mechanism was reported by Nasution *et al.*^36^

## Conclusions

Copolymer nanocomposites based on poly(aniline-*co*-indole) with varying copper alumina contents were developed using an efficient *in situ* chemical oxidation technique. The successful packing of Cu–Al_2_O_3_ nanofillers in the copolymer was confirmed by FT-IR spectra. The XRD studies revealed the existence of an alliance of organic and inorganic fragments in the composite material. The HR-TEM images showed the distribution of nanosized Cu–Al_2_O_3_ fillers in the copolymer and the better dispersion of nanofillers was observed at 5 wt% Cu–Al_2_O_3_ loading. The TG studies confirmed that the inclusion of nanoparticles imparted remarkable thermal stability to PANI-*co*-PIN and the stability of the composites increased with the loading of Cu–Al_2_O_3_ nanoparticles. The PANI-*co*-PIN/Cu–Al_2_O_3_ nanocomposites evinced excellent AC conductivity and dielectric properties. Better electrical traits were observed for PANI-*co*-PIN/5 wt% Cu–Al_2_O_3_ nanocomposite indicating the consistent covering of Cu–Al_2_O_3_ in the copolymer. The excellent ammonia gas sensing property of the 5 wt% Cu–Al_2_O_3_ composite was the combined effect of the interaction of nanocomposites and the excellent p–n junction formed in the material. The combination of high electrical conductivity, dielectric constant, gas sensing and thermal stability makes PANI-*co*-PIN/Cu–Al_2_O_3_ nanocomposites a potential candidate for concocting nano-electronic applications.

## Conflicts of interest

There are no conflicts to declare.

## Supplementary Material
